# Extracranial arteriovenous malformations: towards etiology-based therapeutic management

**DOI:** 10.1172/JCI172837

**Published:** 2025-03-17

**Authors:** Julien Coulie, Emmanuel Seront, Miikka Vikkula, Laurence M. Boon

**Affiliations:** 1 Center for Vascular Anomalies, Cliniques Universitaires Saint-Luc, University of Louvain, Brussels, Belgium; VASCERN VASCA European Reference Centre, Saint-Luc University Hospital, Brussels, Belgium.; 2 Division of Plastic Surgery,; 3 Human Molecular Genetics, de Duve Institute, and; 4 Institut Roi Albert II, Department of Medical Oncology, Cliniques Universitaires Saint-Luc, University of Louvain, Brussels, Belgium.; 5 WELBIO Department, WEL Research Institute, Wavre, Belgium.

## Abstract

Anomalies during angiogenesis can initiate the formation of arteriovenous malformations (AVMs), characterized by aberrant connections between arteries and veins and fast lesional blood flow. These anomalies can manifest anywhere in the body, including the brain, and they typically appear at birth and evolve alongside growth of the individual. Depending on their location and size, AVMs can induce progressive deformation, chronic pain, functional impairment, and ulceration and pose life-threatening risks such as hemorrhage and organ dysfunction. The primary treatment modalities entail surgical intervention or embolization followed by surgery. However, these approaches are often challenging and seldom offer definitive resolution. In addition, inadequately performed surgery may trigger angiogenic rebound, fostering AVM recurrence. Advancements in comprehending the molecular pathways underlying AVMs have sparked interest in repurposing targeted therapies initially devised for cancer treatment. The first results are promising, giving new hope to the patients affected with these often devastating and debilitating lesions, the management of which presents major clinical challenges.

## Introduction

Arteriovenous malformations (AVMs) represent rare and enigmatic vascular anomalies, occupying a distinctive niche within the spectrum of vascular lesions. They arise during vasculogenesis and/or angiogenesis, manifesting as fast-flow lesions characterized by abnormal connections between arteries and veins, bypassing the capillary network. AVMs can appear anywhere on the body, but are more frequently found in the head and neck region. Most AVMs are found intracranially, either in brain parenchyma, or as pial or dural arteriovenous fistulas (AVFs), or combined with deep central vein anomalies (vein of Galen aneurysmal malformation [VGAM]). This review will focus on extracranial AVMs.

The exact incidence of AVMs remains unknown, primarily due to their rarity and the lack of comprehensive registries. Incidence has only been studied for intracranial AVMs. However, historical data approximate the incidence of extracranial AVMs ranging from 5 to 613 per 100,000 of the population (1, 2). Most AVMs occur intracranially, involving the brain structure, and are up to 20 times more frequent than extracranial AVMs (3, 4). It is essential to recognize that such figures may vary significantly across different populations and ethnic groups.

AVMs are thought to be congenital, arising from developmental errors during vasculogenesis (5). Although the congenital nature of some AVMs cannot be denied, as observed in extensive pediatric peripheral lesions, not all AVMs may have a prenatal origin. Animal models have shown that postnatal activation of *KRAS* mutations in endothelial cells (ECs) may cause AVMs (6). Despite their sometimes congenital nature, AVMs can remain asymptomatic for extended periods and diagnosis may be delayed until later stages of life. Trauma or hormonal changes during puberty may unveil their presence. Observations of secondary central nervous system AVFs after venous thrombosis have been published (7, 8). Whether some extracranial AVMs may appear spontaneously over time remains unelucidated.

Typically sporadic and solitary, AVMs can affect any tissue or organ, progressively enlarging over time. Depending on their size and location, they may induce deformation, severe pain, chronic anemia, or functional limitations. Complications can escalate to hemorrhage, tissue ischemia, vascular steal syndrome, or high-output cardiac failure due to rapid blood flow (2, 9). Management necessitates the expertise of a multidisciplinary specialized center. Endovascular embolization followed by surgical resection represents the preferred treatment modality, contingent on AVM angioarchitecture and operator proficiency. However, these interventions present important challenges: (a) they require extensive tissue resections and complex reconstructions; (b) they can be associated with major complications, such as arterial occlusion or tissue necrosis; (c) recurrence rates are high, exceeding 50% within a year after resection (mainly in case of partial resection or in advanced stages) or embolization (4, 10); and (d) incomplete procedures may spur AVM expansion via angiogenic pathways. There is thus a need to improve our comprehension of the pathogenesis of AVMs to develop new therapeutic approaches, including precision medicine with targeted therapies, to treat patients not suited for embolization or surgery and to be evaluated as (neo-)adjuvant therapy.

## Clinical presentation and evolution

**Figure 1:**
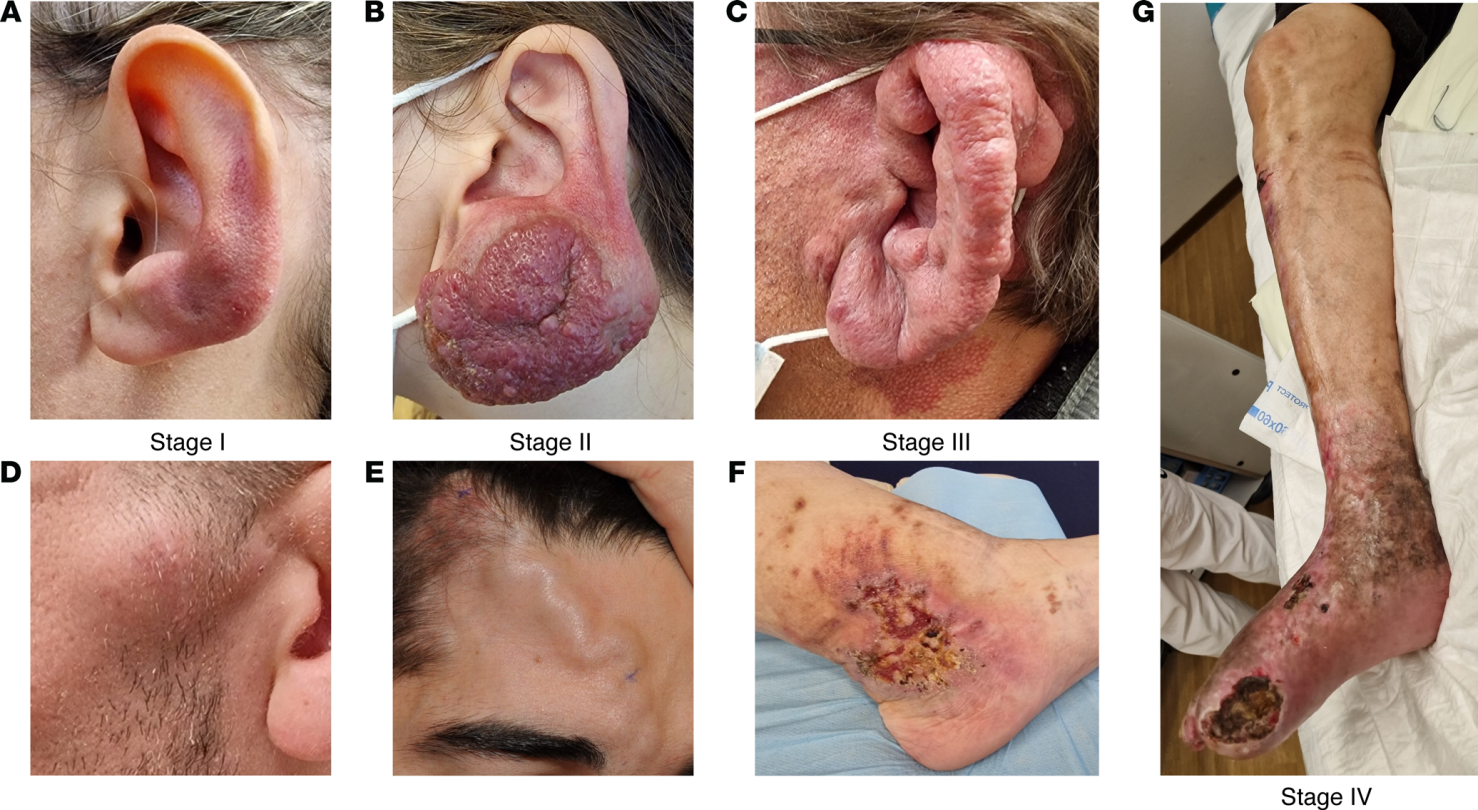
Clinical aspects of AVMs illustrating the Schöbinger’s staging system. Stage I: cutaneous blush with warmth; a localized left ear AVM (**A**) and a left temporal AVM (**D**). Stage II: bruit, audible pulsations, expanding lesion; a growing left earlobe AVM (**B**) and a right frontal AVM with an important glabellar draining vein (**E**). Stage III: pain, ulceration, bleeding, infection; a left ear AVM causing pain and severe deformation (**C**) and an ulcerated left ankle AVM (**F**). Stage IV: cardiac failure; an extensive ulcerated AVM of the entire right lower limb causing cardiac insufficiency and pulmonary hypertension (**G**). All photos are shown with patient consent.

AVMs exhibit a diverse range of clinical presentations, largely contingent upon their anatomical location and tissue involvement. Patients may present with pain, swelling, redness (Figure 1, A–C), and a palpable thrill, a vibratory sensation felt on the skin, indicating a fast-flow vascular lesion (Figure 1, D and E). These signs raise suspicion of an AVM. Moreover, as these lesions progress, they may lead to more severe systemic effects, such as ulceration or heart failure (Figure 1, F and G), due to the increased circulatory demand imposed by arteriovenous shunting.

Extracranial AVMs are mostly isolated but can be part of an inherited disease in the context of a germline mutation in up to 30% of cases (11). Extracranial visceral AVMs, mainly in the lung or liver, are frequently seen in hereditary hemorrhagic telangiectasia (HHT) and can be observed in capillary malformation–AVM (CM-AVM). Outside of the abdomen, extracranial AVMs most commonly affect the head and neck area (50% of extracranial AVM), followed by extremities. Fifty percent of head and neck AVMs affect the oral and maxillofacial region; a retrospective review showed that cheek and ear were involved in 31% and 16% of patients with head and neck AVM, respectively (12, 13).

The morphological presentation of extracranial AVMs can vary widely, ranging from localized AVFs to diffuse tissue involvement. Localized forms can present as a single AVF or single nidus with one or more feeding arteries. Others appear as hypervascularized masses or hemangioma-like masses (Figure 1B) and others as diffuse infiltrating vessel entanglements (Figure 1F), raising challenges in differential diagnosis (14).

AVMs can infiltrate various tissues, causing a variety of symptoms and presentations. Extracranial AVMs affecting the skin are often warm upon touch with a visible erythema. The fast flow through the malformation can precipitate complications such as ulceration (Figure 1, F and G) and bleeding. Ulceration may arise from local tissue ischemia secondary to vascular shunt or due to localized venous hypertension (5). Mucosal involvement is frequently associated with recurrent bleeding. Primary bone AVMs are found in the maxillofacial region, but notably, they are only found in teeth-bearing bones (12).

Around 45% of AVMs are detected at birth, 20% later during childhood, and another 15% during puberty. Adulthood represents a period where approximately 20% of cases become visible (3). While AVMs are thought to be congenital, external events such as trauma can precipitate their clinical manifestation in about 2.5% of cases, indicating a role for external triggers in exacerbating preexisting conditions (12). Hormonal changes significantly influence the progression of AVMs, with puberty and pregnancy marking periods of first clinical appearance in 10% to 15% and 8% of cases, respectively (3, 12).

### Classification of AVMs.

**Figure 2:**
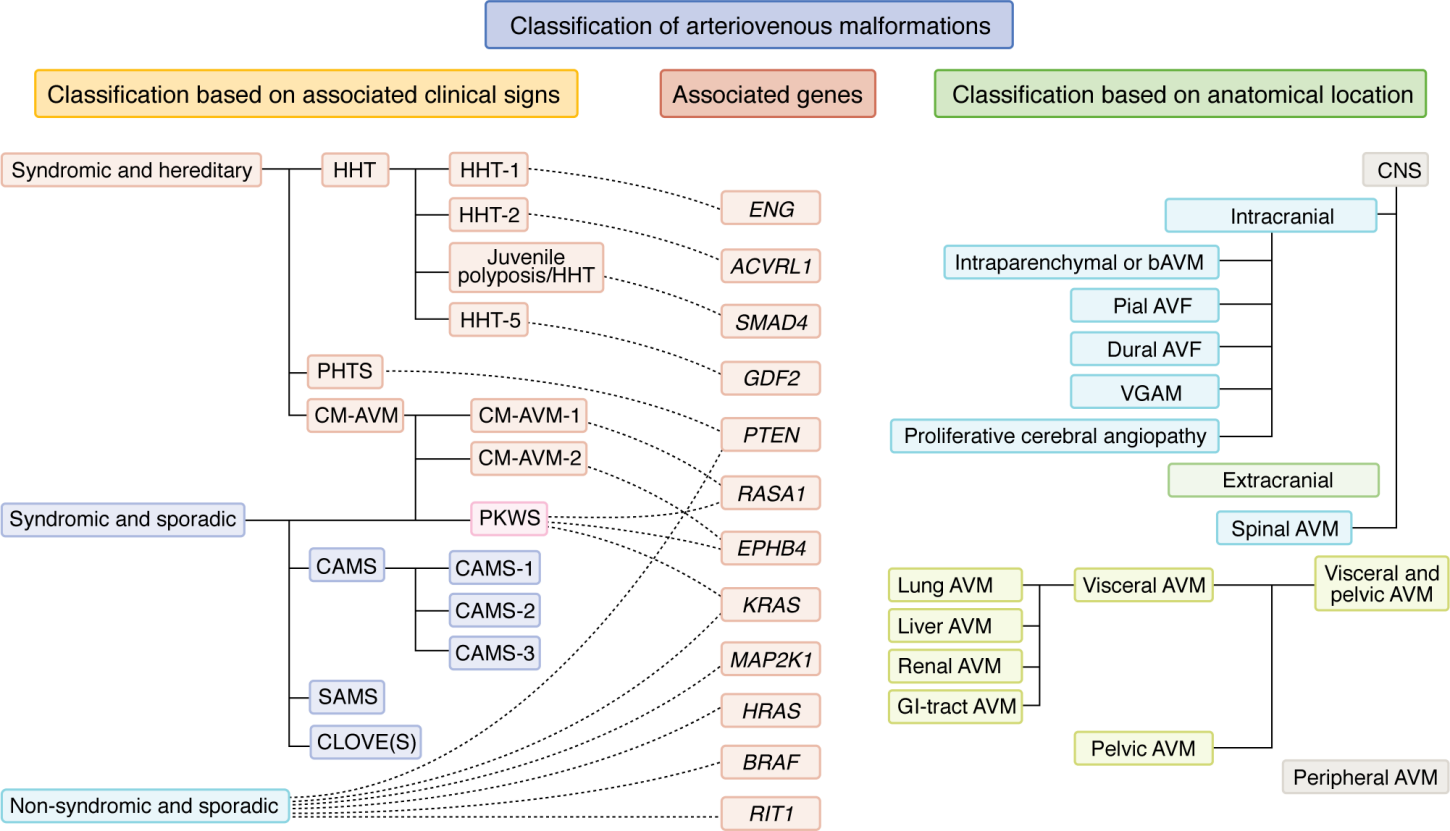
Illustration of AVM subclassification based on either (a) associated clinical signs, (b) associated genes, or (c) anatomical location. Notice the clear correlation between the classification based on associated clinical signs and genes. GI-tract, gastro-intestinal tract. Adapted from ref. 16.

AVMs represent a heterogenous group of fast-flow lesions that can arise anywhere on the body causing a variety of symptoms. The 2018 International Society for the Study of Vascular Anomalies (ISSVA) classification only differentiates AVMs from AVFs, specifying that each one can either be sporadic, part of HHT or CM-AVM (ISSVA Classification of Vascular Anomalies; ref. 15). The European Reference Network for Vascular Anomalies (VASCERN-VASCA) presented a more elaborate classification system as part of a patient pathway (16). It delineates a classification system for AVMs, beginning with a subdivision based on clinical manifestations or anatomical positioning. As summarized in Figure 2, AVMs are categorized into three groups based on associated clinical signs: (a) hereditary syndromic AVMs, (b) sporadic syndromic AVMs, or (c) sporadic nonsyndromic AVMs. Hereditary syndromic AVMs include (1a) HHT, (1b) phosphatase and tensin homolog–associated (*PTEN*-associated) hamartoma tumor syndrome (PHTS), and (1c) CM-AVM. Sporadic syndromic AVMs encompass patients with (2a) Parkes-Weber syndrome (PKWS, that can either present in a sporadic or hereditary form as part of the CM-AVM spectrum), (2b) cerebrofacial arteriovenous metameric syndrome (CAMS), (2c) spinal arteriovenous metameric syndrome (SAMS), or (2d) congenital lipomatous overgrowth, vascular malformations, epidermal nevi and scoliosis/skeletal/spinal anomalies (CLOVE[S] syndrome). Sporadic nonsyndromic AVMs are further classified by location: CNS, either (3a) intracranial or (3b) spinal, (3c) visceral or intrapelvic, and (3d) peripheral. The pathway examines specific clinical indicators, known genetic mutations, and recommends diagnostic modalities for each AVM subtype and will be available on the VASCERN-VASCA website (https://vascern.eu/group/vascular-anomalies/clinical-decision-support-tool-vasca/vasca-patient-pathways).

### Clinical staging systems.

There is a need for staging AVMs, as they progressively aggravate over time. More precise staging should help clinicians decide when and how to treat them and facilitate studies of treatment response. Numerous attempts have been made to propose a clear and concise staging system (17–19), including the Schöbinger classification (12). This system includes four stages, which are established on clinical manifestations and symptoms (Figure 1). While this staging system does not directly incorporate the aspect of progression, AVMs typically worsen in time, generally resulting in an escalation through the Schöbinger stages.

A treatment protocol was aligned with the Schöbinger staging system, by Kohout et al., endorsing surgical interventions or a combination of embolization and surgery for AVMs at stages I and II (12). The authors suggested initiating treatments at stages II and III if the AVMs exhibited a tendency for rapid progression. They did not elaborate specific treatment modalities. They also observed that the management of early-stage AVMs (stage I) is contentious due to the extensive nature of the required resection performed predominantly in young patients, given the unpredictable course of the condition (12). As treatment depends on the location and angioarchitecture of the lesion, another staging system was proposed, which combines clinical and radiologic information as well as a treatment algorithm (20). It is currently under review to be modified to include genetic mutations and the Yakes angioarchitectural classification (21).

## Standard management

There is no consensus on the best therapeutic management of AVMs. Most authors argue for surgery and/or endovascular therapy with the goal to either remove or destruct completely the nidus. Some favor follow-ups, especially for early stage lesions. Other therapeutic options have also been described, and antiangiogenic medications are emerging. Depending on the center and their expertise, complete surgical resection, usually with preoperative embolization or endovascular embolization and endothelial nidus destruction seem to offer the best results. Yet recurrences are frequent, underscoring extracranial AVMs as the most difficult vascular malformations to treat. Most authors agree that no remission can be considered definite before 5 years after treatment, yet about 5% of extracranial AVMs recur 10 years after treatment (22).

The therapeutic objective of surgery when treating an AVM is total resection with margins to excise the nidus and manage the malformation, as a partial resection can worsen the AVM. Surgery is recommended for small, localized AVMs to prevent severe sequelae or disfigurement (22, 23). Staged surgical resections are debated due to risk of recurrence between operations (23). Preoperative embolization does not reduce recurrence, but helps in differentiating the nidus and minimizing perioperative blood loss (18, 23). Surgical outcomes show varying recurrence rates influenced by resection accuracy, with figures as high as 85% in certain series (22, 23). Residual blood flow and incomplete removal are major recurrence factors (24). Postoperative reconstruction for contour or functional deficits may employ free flaps to potentially decrease recurrence by minimizing local hypoxemia and angiogenic response, though this benefit is not universally accepted (25, 26).

Endovascular treatment of AVMs aims to obliterate blood flow and destroy the endothelium within the nidus, as mere blood flow blockage can result in revascularization. Multiple treatments should be closely staged to reduce recurrence. All AVMs can undergo endovascular treatment, but prior arterial ligation may impede further endovascular access. Preoperatively, embolic agent selection is crucial, with options including particles, sclerosing liquids, polymerizing glues, and mechanical occlusive devices, the latter of which is insufficient alone (27). Transarterial, transvenous, and direct transcutaneous nidus puncture are the primary endovascular access methods, with the choice depending on specific lesional requirements and/or the operator’s expertise. Efficacy rates, as reported by various authors, range from 80%–100% in terms of angiographic improvement (23, 27), although complete eradication is rare, occurring in only 7%–10% of cases (23, 27). Treatment efficacy is linked to the experience of the physician and the thoroughness of nidus devascularization and endothelial destruction as well as the embolization technique and agent that is used. Approaches and techniques used depend on the angioarchitecture of the lesion, as underlined by numerous radiological classification systems (21, 28). Awareness of the risk of paradoxical embolization and systemic effects of embolic agents is essential for optimizing outcomes.

## VEGF plays a key role in arteriovenous differentiation

Angiogenesis is a complex process regulated by different signaling pathways that responds to numerous stimuli. Vascular endothelial growth factor (VEGF) is a potent EC mitogen, driving angiogenesis through various signaling pathways, including the phospho-inositol kinase 3 (PI3K)/ protein kinase B (AKT)/mammalian target of rapamycin (mTOR) and the MAPK/extracellular regulated kinase (ERK) pathways. Under physiological conditions, activation of these pathways supports EC survival, proliferation, migration, and maintenance of vascular homeostasis by facilitating EC differentiation.

**Figure 3:**
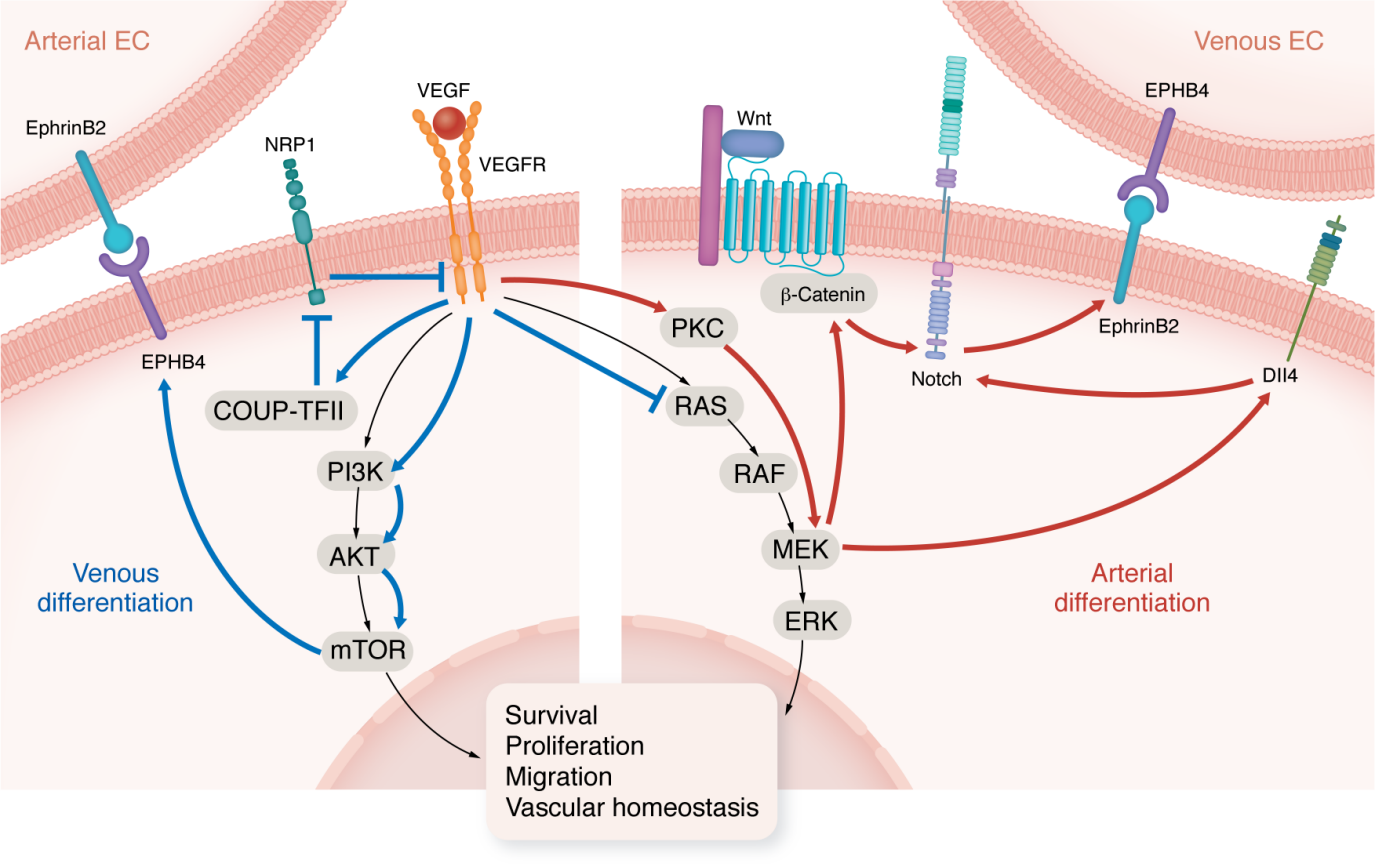
Illustration of the main molecular pathways involved in arteriovenous differentiation. PKC, protein kinase C.

Arterial and venous ECs exhibit distinct responses to VEGF stimulation. Arterial ECs primarily engage the protein kinase C/MAPK cascade to trigger activation of the NOTCH signaling pathway via δ-like ligand 4 (DLL4) and the Wnt/β-catenin pathway. The NOTCH pathway in turn induces expression of arterial markers (EphrinB2). The venous phenotype is sustained through a different response to VEGF stimulation via the expression of chicken ovalbumin upstream promoter-transcription factor II (COUP-TFII), which blocks neuropilin-1 (NRP1), a coreceptor of VEGF–receptor 2 (VEGFR2). This leads to activation of the PI3K/AKT/mTOR pathway and downregulation of the MAPK pathway (30–32), resulting in suppression of EphrinB2 expression, while expression and activity of ephrin type-B receptor 4 (EPHB4) becomes predominant (33), as summarized in Figure 3.

In the context of AVMs, VEGF assumes a crucial role in both development and growth. Preclinical studies utilized mouse models of activin-like kinase receptor-1 (ALK1) deficiency to evaluate this. ALK1 is a receptor for members of the TGF-β family, and its deficiency is associated with excessive expression of VEGF (9). The studies elucidated the indispensable role of VEGF stimulation for inducing AVM formation, resulting in heightened dysplasia index and clustered EC proliferation within dysplastic vessels (2). Elevated VEGF levels in the brains of mice with ALK1 deficiency–induced AVMs have also been linked to increased AVM-related hemorrhage rates and mortality, suggesting that VEGF is not only involved in AVM development, but also in subsequent complications (34). Moreover, clinical reports have documented significantly higher expression levels of multiple proangiogenic factors, including VEGF, in resected sporadic brain AVMs compared with normal brain tissue of epileptic AVM-free patients (35, 36). Plasma levels of VEGF were found to be elevated in some AVM patients compared with healthy controls (37). VEGF may thus serve as a potential biomarker for treatment efficacy, as indicated by a significant reduction in mean plasma VEGF levels after brain AVM resection (38). Finally, VEGF contributes to the high recurrence rate of AVMs following local treatments, such as surgery and embolization. Endothelial VEGF expression is in fact more prevalent in patients with incompletely embolized AVMs, suggesting that invasive treatments may stimulate reactive angiogenesis and EC proliferation in an effort to repair the denudation of vessel walls (39).

## Genetic background of AVMs

The majority of AVMs arise sporadically as isolated lesions. In these noninherited AVMs, the important MAPK signaling cascade plays a crucial and pathogenic role. Signaling via this pathway is initiated by the activation of protein kinase rat sarcoma virus protein (RAS) by cell-surface receptors. Subsequently, RAS triggers the signaling cascade involving rapidly accelerated fibrosarcoma kinase (RAF) and MAP-extracellular signal-regulated kinase 1 (MEK1), culminating in the phosphorylation and translocation of ERK1 and 2 into the nucleus. Once in the nucleus, ERK activates various transcription factors (such as c-FOS, c-JUN, and c-MYC), pivotal for regulating cell proliferation and metabolism.

In extracranial AVMs, somatic mutations in *MAP2K1*, encoding MEK1, have been detected in 64% of patients (40). These mutations, typically missense or small in-frame deletions, affect the negative regulatory domain of MEK1, resulting in its increased activity (38). *BRAF*, *KRAS*, and *HRAS* mutations have also been described in extracranial AVMs (41–43). These mutations may cause distinct phenotypes. For instance, in the facial region, *KRAS*-mutated AVMs seem to be associated with more severe and extended clinical presentations than *MAP2K1-*mutated AVMs (41, 44).

Experimental studies utilizing mouse models have provided compelling evidence supporting the role of activated KRAS within ECs in the development of AVMs across various soft tissues, including the brain, liver, and heart. Furthermore, treatment with the mitogen activated protein kinase (MEK) inhibitor trametinib was associated with improved survival rates and normalization of vessel sizes and morphology, emphasizing the therapeutic potential of targeting the MAPK pathway in AVM management (45). The prevalence of MAPK pathway mutations, particularly *KRAS* mutations, in cerebral AVMs, along with experimental evidence from mouse models and therapeutic efficacy of MEK inhibition, collectively support the pivotal role of the MAPK cascade in the pathogenesis of AVMs.

Inherited AVMs are caused by loss-of-function mutations in proteins affecting the MAPK pathway (RAS p21 protein activator 1 [*RASA1*]*/EPHB4*), phosphatase and tensin homolog (*PTEN*), or the TGF-β/ bone morphogenic protein (BMP) pathway (endoglin [*ENG*], *ACVRL1*, *SMAD4*, *GDF2*/*BMP9*).

### CM-AVM (OMIM 608354).

CM-AVM manifests with multiple small capillary malformations, many of which have reduced resistance index by Doppler ultrasound, and which can be surrounded by a pale halo, primarily localized on the face and limbs. AVMs are observed in 18%–24% of CM-AVM cases, typically manifesting in the skin, muscle, bone, spine, and brain. These lesions can lead to life-threatening complications, such as bleeding, congestive heart failure, and neurological consequences. CM-AVM encompass two subtypes:

### CM-AVM1.

This is caused by inactivating mutation of *RASA1*, a gene encoding a negative regulator of RAS in approximately 70% of cases. Truncating mutations in *RASA1* have been described, with instances of somatic second-hit mutations identified in affected limbs of PKWS patients, a limb overgrowth associated with CM and multiple AVFs (44, 46, 47). These patients also have multiple CMs on the rest of the body.

### CM-AVM2.

This results from truncating mutations or amino acid substitutions in *EPHB4*, leading to its diminished or absent expression, termed haploinsufficiency. EPHB4 interacts with RASA1 to inhibit RAS/MAPK signaling; loss-of-function mutations in these genes lead to sustained activation of MAPK. It also plays an important role in RAS/MAPK/ERK signaling regulation through its interaction with ephrinB2 (48). CM-AVM2, in contrast to CM-AVM1, can present with telangiectasias and bier spots.

### PHTS (OMIM 158350).

*PTEN* encodes a critical protein serving as a tumor suppressor, which plays a pivotal role in regulating AKT activation by PI3K. Germline mutations in *PTEN* are associated with Bannayan-Riley-Ruvalcaba syndrome (BRRS) and Cowden syndrome (CS), collectively referred to as PTEN hamartoma and tumor syndrome (PHTS). Individuals harboring *PTEN* mutations may manifest various clinical features, including vascular anomalies, such as AVMs (49). A retrospective analysis involving 26 patients with *PTEN* mutations revealed that 14 individuals (54%) exhibited vascular anomalies, with 12 displaying fast-flow vascular malformations. The observed spectrum of findings ranged from infiltration of the affected tissue by fine, tortuous arterial and venous channels accompanied by tissue blush to direct AVFs resulting in significant enlargement of proximal draining veins. Notably, the angioarchitecture of these vascular anomalies differed from that of *PTEN* WT AVMs, characterized by distinctive segmental dilatation of draining veins. Furthermore, the involvement of multiple noncontiguous sites, observed in 57% of *PTEN*-mutated patients, represents an unusual feature rarely seen in individuals with non-syndromic AVMs (50).

### HHT (OMIM 187300).

Characterized by subcutaneous telangiectasias and AVMs in various organs, HHT is primarily caused by mutations in TGF-β/BMP signaling pathway genes, including *ENG* (HHT1), *ACVRL1* (ALK1; HHT2), *SMAD4* (juvenile polyposis and HHT syndrome), and *GDF2* (HHT5). ENG and ALK1, predominantly expressed on EC surfaces, bind TGF-β/BMP ligands, facilitating downstream signaling involved in angiogenesis and vascular remodeling. Loss-of-function mutations in these genes destabilize vascular structures and enhance disorganized angiogenesis (51–55). Phenotypic variations exist among HHT types, with HHT1 typically presenting more severe symptoms, often affecting the lungs and brain, and HHT2 more frequently involving the liver (56, 57). HHT was reviewed in the *JCI Vascular Malformations* Review series 2024 (58).

## Targeted therapies in AVM

**Figure 4:**
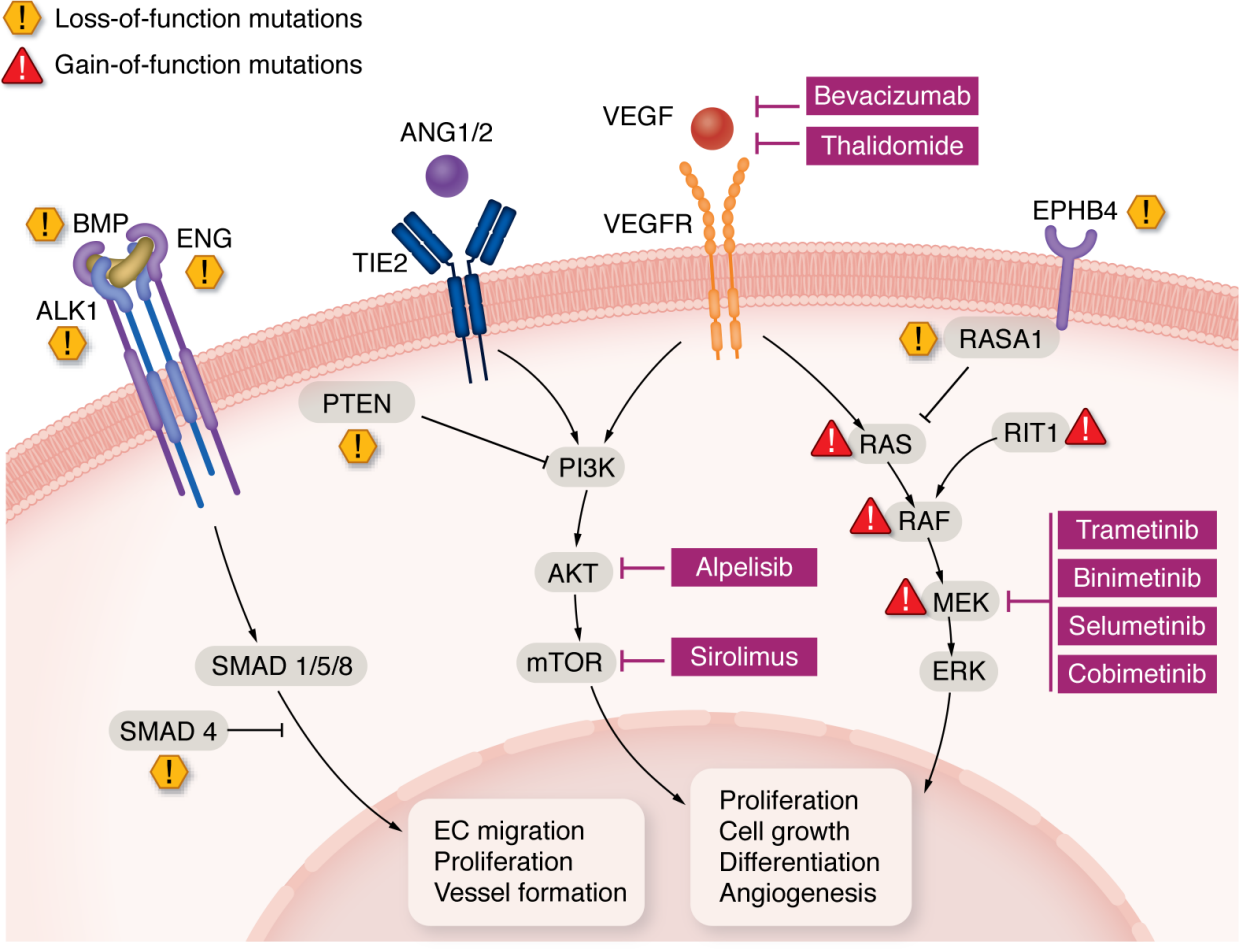
Illustration of the main pathways involved in AVMs and their functions in vascular development and maintenance. Selected inhibitors of these pathways are shown in magenta boxes. Red triangles indicate hyperactivating (gain of function) somatic mutations, as seen in nonsyndromic sporadic AVMs. Yellow hexagons indicate loss-of-function mutations responsible for syndromic hereditary AVMs. SMAD, fusion of Caenorhabditis elegans Sma genes and the Drosophila Mad, Mothers against decapentaplegic; RIT1, Ras like without CAAX 1.

The unraveling of the major initiating mechanisms for AVM formation has propelled the field to think of targeted molecular therapies (Figure 4) that could be used alone or as pre-, peri-, and postinterventional procedures.

### Targeting the VEGF pathway.

Given the pivotal role of VEGF in AVM development, targeting VEGF signaling emerges as a compelling strategy to manage AVMs. Bevacizumab, a monoclonal human anti-VEGF antibody, has demonstrated efficacy in reducing the proliferation of vascular cells, vessel density, and dysplasia index in mice with ALK1-induced brain AVMs (35). However, the results of clinical case reports of bevacizumab efficacy on AVMs have been unsatisfactory. In a single-arm pilot study, two patients with large, deemed unresectable brain AVMs received bevacizumab (5 mg/kg every 2 weeks for 12 weeks) without any significant change in AVM volume (59–61).

An ongoing phase II–III, placebo controlled, clinical trial is evaluating the efficacy and safety of intravenous bevacizumab in patients with symptomatic cerebral AVMs (BevacizuMAV; ClinicalTrials.gov NCT06264531). Although small tyrosine kinase inhibitors targeting the VEGF receptor (VEGFR) are currently being evaluated for HHT, no trial to date has assessed their efficacy in sporadic extracranial AVMs. Bevacizumab has demonstrated more significant efficacy in treating HHT, as shown in various clinical trials. This effectiveness was first observed anecdotally in HHT patients receiving bevacizumab for cancer treatment, during which their HHT symptoms unexpectedly improved (62, 63).

### Immunomodulatory imide drugs.

Thalidomide exhibits potent antiangiogenic effects by various mechanisms inhibiting cytokines like FGF and VEGF, as well as capillary microvessel formation and EC migration. Thalidomide, by binding to cereblon, a E3 ligase adapter, recognizes some proteins such as VEGF, and facilitates their degradation through the ubiquitin/proteasome pathway (64). Furthermore, thalidomide has been shown to reduce the protein expression of angiopoietin-2 (ANGPT-2) and VEGF as well as to decrease mRNA expression of ANGPT-2 in patients with Crohn’s disease, a condition characterized by elevated levels of these proteins. Through this mechanism, thalidomide significantly inhibited cell proliferation, cell migration, and capillary-like tube formation in human umbilical vein ECs (HUVECs). These findings may explain the antiangiogenic efficacy of thalidomide (65).

Thalidomide has strong antiinflammatory and immunomodulatory properties by targeting TNF-α and nitric oxide. In a mouse model of brain AVM, thalidomide and its derivative lenalidomide reduced dysplastic vessels and hemorrhage while increasing mural cell coverage, possibly through increased PDGF-β expression. These effects were accompanied by a reduction in CD68^+^ cells and inflammatory cytokines in murine cerebral AVM lesions (66). Building upon these findings, a prospective clinical study was conducted evaluating thalidomide in 18 patients with extensive, recurrent, and highly symptomatic extracranial AVMs refractory to conventional treatments (67). An initial dose of 50 mg thalidomide daily was escalated to 100 or 200 mg daily within two weeks for the first five patients. Subsequent patients received a continuous lower dose of 50 mg daily due to grade 3 adverse events (asthenia, erythroderma, and small cerebral infarct) observed in four of the patients receiving the higher dose. Thalidomide led to significant clinical improvement in all patients, including pain reduction, cessation of bleeding, and healing of ulcerations. Cardiac overload that was present in three patients resolved. Notably, thalidomide induced angiographic reduction in two patients and disappearance of AVMs in another one, with sustained effects even after discontinuation. In a similar way, thalidomide was shown to reduce the severity and frequency of epistaxis in patients with HHT, increasing the levels of hemoglobin. In vivo studies showed that thalidomide treatment was able, through increased PDGF-B expression in ECs, to stimulate mural cell coverage (68). Recently, pomalidomide demonstrated a superior efficacy compared to placebo in patients with HHT in significantly reducing epistaxis severity (69).

Thalidomide may also be promising in association with or following embolization and/or surgery. In this study, thalidomide was associated with embolization in seven patients (two from the high-dose and five from the low-dose group). Compared with the previous history and efficacy of embolization alone on these patients’ lesions, the association of embolization with thalidomide allowed a more efficient and durable efficacy. Embolization can, by denudation of the vessel wall surface, trigger hypoxia-inducible transcription factor (HIF) signaling and subsequently stimulate angiogenesis by release of VEGF, which also enhances the intrinsically high cytokine activity in the AVM, causing an increase in the level of inflammation (45). Thalidomide, by targeting angiogenic pathways as well as the inflammatory reaction, may potentialize the effect of embolization compared with embolization alone.

Thalidomide is generally well tolerated, with manageable toxicity. The most common side effects were mild fatigue and polyneuropathy, which resolved upon discontinuation of the medication. While thalidomide has been associated with an increased risk of thrombotic venous events in cancer patients, this risk does not seem to be significantly elevated in vascular anomaly patients, as seen in this study and shown in an HHT series. Nonetheless, caution is warranted, as one case of fatal nose bleeding occurred in an HHT patient receiving thalidomide, indicating a potential risk of vessel wall destabilization and increased bleeding risk (70, 71). Thalidomide derivatives appear as promising alternative agents due to their better toxicity profile. A large metaanalysis comparing thalidomide and lenalidomide in myeloma reported that the discontinuation rate from thalidomide trials was higher than that from lenalidomide trials, due to the toxicity profiles (72).

### Targeting the PI3K/AKT/mTOR pathway.

Given the interplay between the RAS and PI3K pathways, mTOR inhibitors have also been trialed. Some clinical benefits have been observed, but significant responses have not been consistent (23, 73). In a retrospective analysis, the efficacy of sirolimus was assessed in ten patients with extracranial AVMs, including seven children (73). The administered sirolimus doses ranged from 0.6 to 3.5 mg/m^2^, with a median treatment duration of 24.5 months (ranging from 4.5 to 35 months). Among the patients, five exhibited no response to sirolimus treatment, while the remaining five showed a partial response, observed at a median time of three months (interquartile range [IQR]: 1; 5), reflecting a limited efficacy of sirolimus in the management of extracranial AVMs. In another retrospective trial including four patients with AVM, sirolimus was not efficient (74). Considering the specific vascular pathways involved, mTOR inhibitors may be best suited for *PTEN*-mutated AVMs. Some case reports or series including *PTEN*-related AVMs showed improvement in symptoms of vascular anomalies, induced by sirolimus (75–78). However, further research is needed to confirm this.

### Targeting the MAPK pathway.

As previously discussed, the MAPK pathway is frequently dysregulated in AVMs. Preclinical investigations have demonstrated that the MEK inhibitor trametinib can enhance survival and normalize vessel morphology in mice with the *KRAS* mutation (6). Trametinib is an oral, reversible, and highly selective MEK1/2 inhibitor. Two clinical cases demonstrated the potential efficacy of trametinib in treating AVMs. A *KRAS*-positive AVM of the chest and spinal cord was treated with trametinib for up to 2.5 years with a subsequent reduction in size and in lesional blood flow rate (79). Moreover, a 16-year-old girl with CM-AVM2 and *EPHB4* mutation was successfully managed using trametinib (80). This patient presented with multiple Bier spots, painful limb overgrowth, and heart failure. Although trametinib was initiated at 2 mg daily, it was swiftly reduced due to skin toxicity, a well-known related adverse event. Despite the lower dose of 0.5 mg daily, cardiac function improved, and leg pain decreased without diminishing hypertrophy.

At the ISSVA World Congress 2022, we unveiled preliminary findings from our TRAMAV clinical trial, a prospective single-center study (EudraCT: 2019-003573-26) (81). Trametinib showed promising efficacy in the ten patients with extracranial AVMs. However, acneiform rash became a notable concern, particularly in the first two patients who began treatment with a daily dose of 2 mg and developed grade 3 skin toxicity, prompting us to adjust the dosage. Trametinib resulted in significant symptom improvement in eight patients, alleviating bleeding, pain, and ulceration. The second part of the TRAMAV trial will focus on evaluating trametinib in children, where skin toxicity may be less pronounced owing to their prepubertal status, thus shedding further light on its therapeutic potential in this population. Other MEK1/2 inhibitors have been approved by the FDA for oncologic indications, including binimetinib and selumetinib, and they may also be active in AVMs. Cobimetinib is currently being evaluated in a phase II trial enrolling patients with extracranial AVM (COBI-AVM study; NCT05125471). None of the molecules discussed here are currently approved for AVMs, underlying the important need to develop medical management for vascular anomalies.

### Finding the right dose.

Interestingly, the doses of targeted agents like bevacizumab, thalidomide, and trametinib required to effectively treat AVMs are notably lower than those used in cancer therapy. As lesion disappearance or strong size reduction has rarely been seen in vascular anomaly patients treated with medications, the goal now rather is to reach the maximal reduction in signs and symptoms with the smallest possible dose to reduce side effects. By precisely targeting these driver cascades at lower doses, treatment remains effective while reducing toxicity, allowing for longer treatment durations with fewer side effects.

### Finding the right drug.

AVMs and certain cancers share hyperactivated molecular pathways, occasionally involving identical hotspot mutations. This overlap opens the door for the repurposing of oncologic drugs to treat AVMs. Many cancer treatments targeting these dysregulated pathways are well characterized, and we reviewed some that have already been explored in the context of AVM management. Advances in the understanding of AVM pathophysiology, supported by in vitro studies, animal models, and genetic testing, combined with knowledge of side effect profiles from cancer therapies facilitate the faster transition of these agents into clinical trials for AVM patients. Unlike cancer, AVMs represent chronic conditions, meaning that any pharmacologic therapy, unless curative, would require prolonged administration. This extended use increases the likelihood of cumulative toxicity or the exacerbation of side effects, presenting a challenge for long-term tolerability. Therefore, the future of AVM treatment may lie in a multimodal approach, integrating drug therapies with ablative techniques, such as embolization or surgical resection, mirroring the combinatory strategies often employed in cancer management. Moreover, novel tools such as vascular organoids that would express one of the AVM-associated pathogenic variants, e.g., the frequent hot-spot changed in *KRAS*, could be used as models for high-throughput screens, at least for the already FDA-approved drugs (82).

## Conclusion

The advancement of targeted therapies holds promise for improving outcomes in patients with AVM. However, individual efficacy varies, and complete cure remains elusive, often accompanied by limiting toxicities. Future trials are essential to identify both clinical and genomic features that can predict treatment efficacy, as well as the optimal duration. We also need to study the sequencing of these therapies relative to other standard modalities, such as surgery and embolization.

## Author contributions

JC wrote the original draft of the manuscript. ES reviewed and edited the manuscript. MV reviewed and edited the manuscript, acquired funding, and did project management. LMB reviewed and edited the manuscript, acquired funding, and did project management.

## Version 1 03/17/2025

Electronic publication
